# Reply to Trela, B.A.; Guffroy, M.R. Comment on “AlAsmari et al. Venetoclax Induces Cardiotoxicity Through Modulation of Oxidative-Stress-Mediated Cardiac Inflammation and Apoptosis via NF-κB and BCL-2 Pathway. *Int. J. Mol. Sci.* 2022, *23*, 6260”

**DOI:** 10.3390/ijms27146166

**Published:** 2026-07-10

**Authors:** Abdullah F. AlAsmari, Adel Alghamdi, Nemat Ali, Fawaz Alasmari

**Affiliations:** Department of Pharmacology and Toxicology, College of Pharmacy, King Saud University, P.O. Box 55760, Riyadh 11451, Saudi Arabia; 441105941@student.ksu.edu.sa (A.A.); nali1@ksu.edu.sa (N.A.); ffalasmari@ksu.edu.sa (F.A.)

We are writing this letter to respond to the letter to the editor regarding the comments that were raised [1]. We support and encourage scientific discussion as part of our participation in the scientific community to clarify any misunderstanding and/or misinterpretation.

In our published study, we provided all the necessary information to the scientists and researchers to make it much more convenient for them to reconstruct the study. However, due to many rounds of revisions at the time of submission and the handling of many projects at the same time, the risk of writing insufficient information regarding the methodology is high. Therefore, we are writing this response to clarify any misunderstanding and/or misinterpretation.

- Regarding the cited references, we would like to emphasize that all the provided references reported cardiac adverse events (AEs) in their studies. For instance, Davids and colleagues reported in Table S4 that acute myocardial infarction and cardiogenic shock were among the reported AEs that led to dose reduction after 365 days of therapy in four patients [2]. Furthermore, in the same table (Table S4), the authors reported that acute myocardial infarction (*n* = 1), cardiogenic shock (*n* = 1), and myocardial ischemia (*n* = 1) were among the AEs that led to dose interruption after 365 days of therapy. Nonetheless, in Table S5, they reported that one patient interrupted VTX more than once due to the same AE, which was cardiac ischemia [2]. Although there is not enough information regarding these cases, reporting these AEs after using VTX is noteworthy [2]. In a recent study, the authors revealed that their large cohort analysis revealed that LV dysfunction, atrial fibrillation, myocardial infarction, congestive heart failure, and pericardial effusion were the cardiovascular AEs that were observed for VTX and ibrutinib [3]. In another study, however, the authors described cardiac arrhythmia as one of the most common clinical presentations of TLS (tumor lysis syndrome), and they stated that three patients experienced clinical TLS and seven had clinical parameters of TLS. Furthermore, they described that two patients experienced clinical TLS after the initiation of VTX at a dose of 50 mg in a phase Ib trial, and one of them died [4]. In addition to these references, recently published studies have reported different cardiac adverse events (AEs) in response to VTX [5,6,7]. Indeed, Johnson I.M. and colleagues investigated cardiac events and their prevalence in acute myeloid leukemia (AML) patients treated with VTX plus hypomethylating agents (HMAs) [5,6]. In this study, they reported that 34 out of 170 patients (20%) experienced a total of 48 cardiac AEs, of which 4 patients (12%) had no cardiovascular risk factors and 11 patients (32%) had no previous or preexisting cardiac disease [5,6]. Of all the reported cardiac AEs, a decrease in LV ejection fraction was the most frequently reported AE in 10 patients (21%), followed by an elevation in troponin in 7 patients (15%). In this study, the authors argued that the higher incidence of cardiac AEs in VTX plus HMA patients is not reflective of AEs in older adults, as they compared the cardiac AEs to those observed in elderly AML patients using HMA alone. Furthermore, they reported that the rates of cardiac AEs were lower in the HMA monotherapy group compared to the VTX plus HMA group, although the HMA monotherapy cohort was older than the VTX plus HMA cohort. In another study, Grewal and colleagues recently reported that VTX is associated with high mortality because of cardiac events based on the results obtained from the FAERS (Food and Drug Administration Adverse Events Reporting System) [7]. Indeed, they reported that compared to the ibrutinib, idelalisib, and acalabrutinib groups, the VTX group was associated with higher morbidity and mortality because of cardiac AEs (29.4%). Furthermore, they attributed the high incidence of cardiac arrest in the VTX group to the inhibition of the antiapoptotic protein BCL-2, which is known to induce cardioprotection against ischemia/reperfusion injury [7]. In response to BCL-2, we referred to different references regarding the basic mechanism of the BCL-2 family and how BCL-2, an antiapoptotic protein, is important for cellular defense against insult or stress. It has been reported that apoptosis observed in the heart in response to chronic pressure overload and chronic hypoxia is correlated with an increase in the gene levels of the proapoptotic protein Bax and a decrease in the gene levels of the antiapoptotic protein BCL-2 [8]. However, we include here some other noteworthy references regarding the effect of BCL-2 inhibition, especially on the heart, as per the comment. It is well known that the intrinsic apoptotic pathway can be stimulated by intracellular stresses and/or cytotoxic insults, such as DNA damage, oxidative stress overproduction, growth factor signaling loss, calcium overload, and endoplasmic reticulum stress [8,9,10,11,12,13,14]. Furthermore, it has been documented that BCL-2 overexpression in the heart reduced the number of apoptotic cells and induced an improvement in cardiac function in response to ischemia/reperfusion injury (IR) [15,16,17]. Therefore, based on these findings, we hypothesized that BCL-2 inhibition could be harmful to cardiomyocytes.
- Regarding the methodology, the source of VTX, and the formulation preparation procedures, we purchased VTX from Hangzhou Cherry Pharmaceutical Technology Co. (China, 99.5% purity), and it was dissolved in 5% DMSO in 0.9% NaCl, as described previously [18]. With regard to the independent histopathology evaluation, we included an overall or comprehensive evaluation of the heart sections in the Results section and figure legends. In addition to that, we used a morphological scoring system to semi-quantitatively evaluate the myocardial damage or injury. As we reported in our published study in Section 4.9 (Histopathology), the damage quantification from at least ten areas corresponding to myocardial tissue was graded using the following parameters: nuclear enlargement and inflammation based on a four-score evaluation system (0, histopathological changes = 1–25%; 1, histopathological changes = 26–50%; 2, histopathological changes = 51–75%; and 3, histopathological changes = 76–100%). This procedure was conducted in at least ten random areas in each heart section in three animals from each group at 400× magnification. This method has been used and reported in many published studies [19,20,21,22]. With regard to measuring the cross-sectional area (SCA), we used the methods and procedures that are described in previously published studies [19,23,24,25,26,27]. Indeed, we quantified and measured the cardiomyocytes’ CSA from H&E-stained slides by manually and randomly selecting at least 10 cells per heart section using ImageJ software (NIH, Bethesda, MD, USA, version 8) at 400× magnification. All semi-quantitative evaluation and cross-sectional area measurements of the cardiomyocytes were performed by an expert who was blind to the animal groups. Although we acknowledge that histopathology findings can be treatment-related findings or spontaneous effects, which are commonly reported in rats, we would like to emphasize that the heart samples from all groups, including the control group, were handled and processed using the same procedures, and any changes observed between group were objectively reported by an expert who was blind to the animal groups, as mentioned above.
- Regarding CK (creatine kinase), we agree with that CK has an extensive tissue distribution and is less specific and sensitive to the heart compared to troponin [28,29]. However, the European Society of Cardiology (ESC), the American Heart Association (AHA), the World Heart Federation (WHF), and the American College of Cardiology Foundation (ACCF) task force have reported that myocardial injury can be detected by measuring specific markers, such as cardiac troponin-I or T (cTn) or the MB fraction of CK (CK-MB) [30]. Furthermore, the World Health Organization’s (WHO) definition of myocardial infarction reported that the use of non-specific cardiac markers should be replaced with the MB fraction of CK (CK-MB) or, more preferably, cardiac troponin [28]. Moreover, numerous published studies have used CK-MB as a marker for cardiac damage or cardiac toxicity [31,32,33,34,35,36,37]. Therefore, we used cardiac troponin and CK-MB in our study to measure the effect of VTX on cardiac biomarkers. For the absolute and relative heart weights, we agree that there was a slight but not significant increase in absolute heart weight in the VTX group. Although the significant increase observed in relative heart weight could be related to the reduction in body weight, the possibility of VTX having an effect on relative heart weight cannot be ruled out.

Our study aimed to investigate the cellular and molecular effects of VTX on cardiomyocytes. As the first study to examine the effect of VTX on the heart, we performed different experiments to validate our hypothesis to better evaluate the effect of VTX on the heart. Therefore, the need for future studies to further explore and investigate the effect of VTX on the heart is crucial. We acknowledge that we did not measure cardiac function to better evaluate the effect of VTX on the heart, and this is considered a limitation of the current study, as we reported earlier.

While we acknowledge that interferon gamma (IFN-γ), a proinflammatory cytokine, is not produced by cardiomyocytes, it has been reported that it can promote the cardiomyocyte production of TNF-α (tumor necrosis factor), which is one of the most common and well-studied cytokines involved in cardiac cell damage signaling [38]. Furthermore, different studies have demonstrated the role of IFN-γ in cardiotoxicity and how its blockade ameliorates doxorubicin-induced cardiotoxicity [39,40,41]. Therefore, we measured the expression of different proinflammatory cytokines, including IFN-γ, to investigate the potential role of inflammatory signaling pathways and to better examine the role of IFN-γ and TNF-α in cardiotoxicity in response to VTX.

We hope that this response addressed the questions and comments raised regarding our study. Furthermore, we welcome collaborative efforts to further address any issues raised in regard to Venetocalx (VTX).
